# Validation of Brix refractometer to estimate immunoglobulin G concentration in goat colostrum

**DOI:** 10.14202/vetworld.2021.3194-3199

**Published:** 2021-12-29

**Authors:** Chollada Buranakarl, Sumpun Thammacharoen, Morakot Nuntapaitoon, Sapon Semsirmboon, Kazuo Katoh

**Affiliations:** 1 Department of Physiology, Faculty of Veterinary Science, Chulalongkorn University, Pathumwan, Bangkok, 10330, Thailand; 2 Department of Obstetrics, Gynaecology, and Reproduction, Faculty of Veterinary Science, Chulalongkorn University, Pathumwan, Bangkok, 10330, Thailand; 3 Swine Reproduction Research Unit, Chulalongkorn University, Pathumwan, Bangkok, 10330, Thailand; 4 Division of Functional and Developmental Science of Livestock Production, Graduate School of Agriculture, Tohoku University, Sendai 981-0845, Japan

**Keywords:** caprine, colostrum quality, optical Brix refractometer

## Abstract

**Background and Aim:**

Immunoglobulin G (IgG) concentration is high in goat colostrum, particularly in the first few hours after parturition, and this is important for the kid’s immunity and growth. IgG levels vary depending on several factors, including breed, disease status, colostrum management, handling, and collection time postpartum. A handheld optical refractometer, an affordable instrument that is simple to use in the field, is used widely in dairy farms to measure total solids. However, it can also be applied to estimate colostrum IgG content on the basis of comparison with standard measurement methods, usually radial immunodiffusion. Studies comparing %Brix values in relation to IgG concentration measured using enzyme-linked immunosorbent assay (ELISA) in goats are limited. The present study aimed to evaluate the use of a handheld optical Brix refractometer for the measurement of IgG concentration in goat colostrum, compare results with those using ELISA, and estimate the %Brix cutoff value equating to low-quality colostrum.

**Materials and Methods:**

Colostrum samples were collected on day 0 from 21 goats (nine Black Bengal, six Saanen, and six of their crossbred offspring) and were frozen. Subsequently, they were analyzed for IgG concentration using a goat-specific ELISA test and Brix percentage using a handheld refractometer. The optimum %Brix cutoff value for the evaluation of colostrum quality was evaluated.

**Results:**

The mean IgG concentration and %Brix in colostrum were 10.60±0.64 mg/mL and 25.0±0.9, respectively. There was a significant (p<0.01) correlation between %Brix and IgG concentration. For an IgG concentration of 6.9 mg/dL, the cutoff value for %Brix was 18.5, equating to high specificity (100%) but low sensitivity (50%). A higher %Brix cutoff value of 21.5 showed high specificity (95%) and high sensitivity (100%).

**Conclusion:**

A Brix refractometer can be used to estimate goat colostrum quality with a proposed %Brix cutoff value of <18.5%-21.5% for poor-quality colostrum.

## Introduction

For a few days postpartum, mammalian milk contains high concentrations of nutrients and immunological factors, including immunoglobulin G (IgG), which is essential for newborns. In many breeds of goat, the level of IgG in colostrum has been found to be high, rapidly declining within the 4 days after parturition. As measured using enzyme-linked immunosorbent assay (ELISA), the concentrations of IgG have been shown to decrease in Murciano-Granadina and Majorera goats [1,2]. Our previous study in Black Bengal (BB), Saanen(SA), and their crossbred offspring showed varying levels of IgG. Although IgG concentration differences may appear to vary because of breed [3] or method of measurements [4], the adequacy of IgG content in colostrum is crucial. Colostrum IgG is positively correlated to the growth and survival of newborn kids in many species, such as dairy calves [5], buffaloes [6], and pigs [7]. In addition, the previous studies have demonstrated a positive correlation between serum IgG and both body weight and average daily gain on 21 days in dairy calves [5] and on 30 days in buffaloes [6]. Thus, increased serum IgG in kids was attributed to passive transfer from the mother at first milking. The passive transfer of colostrum IgG in calves induces improved intestinal development with higher weight gain and lower morbidity and mortality rates when compared with those in calves receiving either mammary secretion obtained after the first milking on days 2 and 3 after calving, known as transition milk or milk [8]. Kids that have not received adequate colostrum intake could experience detrimental effects in terms of growth and survival. Other external factors, especially heat, can influence the levels of colostrum IgG content. Heating colostrum may be necessary for preventing the transmission of pathological agents from mother to kids. A previous study in ewes have shown, several factors affect colostrum quality, including parity, length of the dry period, age at first lambing of primiparous ewes, lambing season, and year [9]. Management of colostrum (such as heat treatment) may also affect its quality. One disease, caprine arthritis encephalitis (CAE), an infection caused by a lentivirus, causes economic losses since goats may have a shorter lactation period, lower milk yield, and altered milk composition [10]. The clinical signs include weight loss, chronic arthritis, encephalomyelitis, and mastitis [11]. On farms where some goats have shown a positive serological test for CAE infection, farmers are advised to heat colostrum before use to prevent the passive transfer of the virus. However, this can lower the IgG content. It has been demonstrated that kids fed colostrum treated at 56°C for 30 min had lower serum IgG levels [12], although no effect on body weight was found on 28 days. Hence, measuring IgG in colostrum before use would be beneficial as it would verify the health status of the goats on the farm.

As thestandard method (radial immunodiffusion) of measurement of IgG is difficult and time consuming, the use of a Brix refractometer has begun to be introduced as a more practical way to estimate IgG in the field. Brix refractometer is used to measure the percentage of sucrose in liquids, especially in agricultural products. The equipment is cheap, produces rapid results, and is practical for farm use. The associations between values obtained from Brix or refractometer and levels of IgG in colostrum of sows have been reported previously [13]; ruminants including cattle, sheep, and goats [4,14-16]; and also in serum of horses [17]. In cows, %Brix results correlated well with results of the radial immunodiffusion (RID) method, with high sensitivity and specificity, and with a Brix cutoff value of 23% corresponding to 50 mg/mL IgG (as determined through RID) [18]. However, the IgG content in the colostrum of goats measured using the same method was lower than that in cows, whereas the cutoff points for %Brix in cows and goats were closer (19.3% and 20.7%, respectively) [16]. Even within the same samples, different methods of IgG measurement may produce varying results. A study of colostrum of Saanen goats and crossbreeds showed that the average levels of IgG in colostrum collected a few days postpartum measured using RID and ELISA were 63.4 and 20.7 g/L, respectively [4]. It has been suggested that the cutoff value for IgG concentration in colostrum using commercial ELISA should be taken as 20 mg/mL [16,19]. The inconsistency of results for IgG levels using ELISA suggests that the cutoff value using %Brix should be verified in each farm at which ELISA is performed. A previous study showed much lower levels of colostrum IgG measured using ELISA in BB, SA, and their crosses at our farm, varying between 6.6 and 16.2 mg/mL [20]. Moreover, there is a limit (10% of the total) on the amount of colostrum that the farm can afford to discard for poor quality colostrum.

The aim of this study, then, was to evaluate the feasibility of using a handheld optical Brix refractometer to estimate goat colostrum quality, as determined using IgG content measured through ELISA.

## Materials and Methods

### Ethical approval

The study was approved by Animal Care and Use Committee, Faculty of Veterinary Science, Chulalongkorn University (Protocol No. 1831051).

### Study period and location

The study was conducted from June 2018 to June 2019 at the Chaipattana Foundation’s Black Bengal Goat Domestication Project, Chiangrai Province, Thailand.

### General farm management

Twenty-one dams, including nine BB, six SA, and their six crossbreds were housed in a conventional open housing system and were mated naturally with either SA or BB bucks. The temperature and relative humidity inside ranged from 14.6 to 32.8°C (mean±SD=26.1°C±4.5) and 42-91% (70.5°C±13.1), respectively. Goats were fed 200 g/head/day (07:00 and 18:00 h) with concentrate (Balance 904^®^, Betagro, Bangkok, Thailand) containing 92.01% dry matter and chemical composition of 17.6% crude protein, 4.52% fat, and 14.8% crude fiber per dry matter basis. The roughage including chopped fresh Napier grass (19.62% dry matter and 10.70% crude protein, 1.99% fat, 69.93% neutral detergent fiber, and 46.89% acid detergent fiber per dry matter basis) and Pangola hay (92.51% dry matter, 4.53% crude protein, 1.61% fat, 71.82% neutral detergent fiber, and 45.61% acid detergent fiber) was also given by 3 kg/head/day with the ratio of 2:1 (w/w). All goats were given free access to water. The routine vaccination against foot-mouth disease and the blood collection for negative results of lentivirus (CAE) and brucellosis were routinely performed once a year. The endoparasite was controlled using a variety of antiparasitic agents such as ivermectin and clorsulon (Ivermectin-F®, Boehringer Ingelheim Animal Health UK Ltd., RG12 8YS, UK), closantel (Telcen®, Cenavisa Animal Health, Reus, Spain), and niclosamide (Nicloverm®, Bukaloe Trading Co. Ltd., Bangkok, Thailand) which were rotational prescribed every 2-3 months. The ectoparasite was controlled twice a year using moxidectin (Cydectin®, Virbac (Australia) Pty. Ltd., NSW, Australia).

### Sample collection

Samples of 15-20 mL of colostrum were collected manually from 21 dams through hand milking into disposable plastic tubes. The colostrum was collected within 3 h after parturition. All sample tubes were placed in a freezer at −20°C immediately after collection and stored there until the day of analysis. The proximal composition and IgG content were determined for all samples.

### Analytical procedure

On the day of analysis, each sample was thawed and kept in a water bath at 40°C for 20 min. Before analysis, all colostrum samples were diluted 3-fold with distilled water. Fat, protein, lactose content, and total solids (TSs) were determined using infrared spectrophotometry (MilkoScan FT2, FOSS Electric A/S, Hillerød, Denmark). IgG concentration was determined by a goat-specific IgG ELISA kit (Cat. no. K3231053P, Koma Biotech Inc., Seoul, South Korea) following the manufacturer’s instructions. After thawing, approximately 0.3 mL of colostrum sample was used for %Brix value determinations using a handheld optical Brix refractometer (RHB-32, YHequipment Co. Ltd., Shenzhen, China).

### Statistical analysis

Data were processed using SigmaPlot^®^ version 12.0. (Systat Software Inc, California, USA). Results are presented as mean±standard deviation. The relationships between %Brix values and other variables (IgG, fat, protein, lactose, and TS) were analyzed using Pearson correlation, whereas, for the relationship between %Brix and IgG concentration (ELISA results), simple linear regression was performed. The test performance (sensitivity, specificity, positive predictive value [PPV], negative predictive value [NPV], and accuracy) were calculated using IgG values measured through ELISA as the standard test. An ELISA IgG level of 6.9 mg/mL was selected as the threshold according to the value of the 10^th^ percentile in this study. The receiver operating characteristics (ROC) curve and the area under the curve (AUC) were calculated to estimate the quality of the cutoff points. A probability value of <0.05 was considered statistically significant.

## Results

Colostrum samples were collected from a total of 21 does, nine BB, six SA, and six crossbreed offspring (BB×SA), whose characteristics are shown in Table-1. Bodyweight values were variable because of the use of the two different goat breeds and the ages of crossbreeds were lower than those for the pure breeds in this study. The number of dams with litter sizes of 1, 2, and 3 was 6, 14, and 1, respectively.

**Table-1:** The characteristic of 21 does from that colostrum was collected.

Variables	Mean	SD	Minimum	Maximum
BW (kg)	31.2	6.4	21.0	43.0
Age (months)	53.2	25.5	8.0	117.0
Parity number	4.7	3.2	1.0	13.0
Litter size (kids/litter)	1.8	0.5	1.0	3.0

BW=Body weight, SD=Standard deviation

Table-2 shows the colostrum compositions, including %Brix values. The maximum %Brix value obtained by the equipment was 32% in one sample, with the remaining 20 samples having lower values. The IgG concentrations were 9.7, 12.5, and 10.0 mg/mL in BB, SA, and BB×SA, respectively.

**Table-2:** Composition of goat colostrum.

Parameters	Mean	SD	Minimum	Maximum
Brix (%)	25.0	3.9	16.0	32.0
ELISA IgG (mg/mL)	10.60	2.92	6.65	16.24
Fat (%)	7.19	2.62	3.65	14.51
Protein (%)	11.59	2.41	3.97	14.11
Lactose (%)	3.63	0.35	2.56	4.25
TS (%)	23.38	2.85	16.28	29.84

BW=Body weight, SD=Standard deviation, IgG=Immunoglobulin G, TS=Total solids. ELISA=Enzyme-linked immunosorbent assay

**Figure-1:**
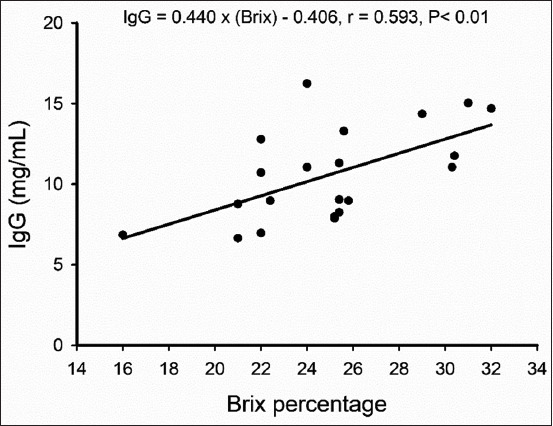
The correlation plot and trend line for %Brix and colostral immunoglobulin G concentrations as measured by ELISA.

Table-3 shows the relationships between %Brix values and other variables in colostrum. Brix percentage positively correlated with IgG concentration (p<0.01) and fat content (p<0.05). However, no correlations were found with protein, lactose, or TS. The regression analysis between %Brix and IgG is shown in Figure-1.

**Table-3:** Pearson correlation coefficients (r) and p- value between Brix and selective variables in colostrum.

Variables	r-value	p-value
IgG	0.593	0.005
Fat	0.444	0.044
Protein	−0.284	0.212
Lactose	0.005	0.983
TS	0.189	0.413

r=Correlation coefficient, P=p-value, IgG=Immunoglobulin G, TS=Total solids

**Figure-2:**
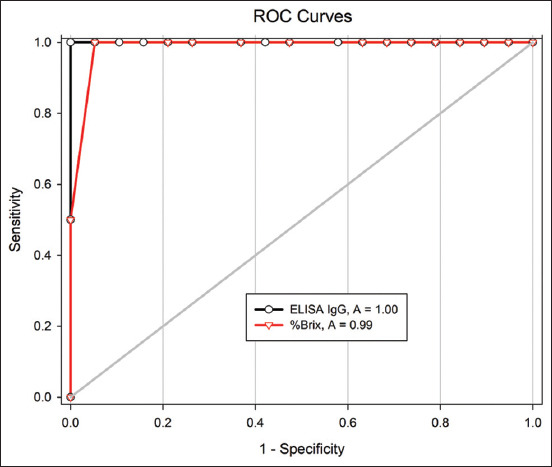
Receiver operator curve comparison of test performance between optical Brix refractometer for the detection of colostrum immunoglobulin G in goat using the poor quality cutoff of 6.9 mg/mL as determined by ELISA test as gold standard.

The cutoff value for an IgG concentration equating to good-quality colostrum was set at 6.9 mg/mL. The AUC for the %Brix readings from the ROC curve was 0.987 (p=0.027) (Figure-2). The cutoff value for %Brix was <18.5, equating to 100% specificity with high accuracy (95.2%) but low sensitivity (50%) (Table-4). NPV was 95.0%, while PPV was 100%. A higher cutoff value for %Brix of <21.5 equated to higher sensitivity (100%) but slightly lower specificity (94.7%). However, PPV was reduced to 66.7%. Values of %Brix >21.5 showed low values for accuracy and PPV.

**Table-4:** Crude accuracy, sensitivity, specificity, PPV, and NPV for different Brix cutoff of goat colostrum using colostrum quality threshold at 6.9 mg/mL IgG as measured by ELISA method for the gold standard test.

Optical % Brix	Sensitivity %	95% CI	Specificity %	95% CI	PPV (%)	NPV (%)	Accuracy	Test high/low
<18.5	50.0	1.3-98.7%	100	82.4-100%	100	95.0	95.2	20/1
<21.5	100	15.8-100%	94.7	74.0-99.9%	66.7	100	95.2	18/3
<22.2	100	15.8-100%	79.0	54.4-94.0%	33.3	100	81.0	15/6
<23.2	100	15.8-100%	73.7	48.8-90.9%	28.6	100	76.2	14/7
<24.6	100	15.8-100%	63.2	38.4-83.7%	22.2	100	66.7	12/9
<25.3	100	15.8-100%	52.6	28.9-75.6%	18.2	100	57.1	10/11
<25.5	100	15.8-100%	36.8	16.3-61.6%	14.3	100	42.9	7/14

PPV=Positive predictive value; NPV=Negative predictive value; Accuracy=Percentage of samples correctly classified has having adequate or inadequate IgG concentration; test high/low=Number of samples declared high (≥6.9 mg of IgG/mL) or low (<6.9 mg of IgG/mL) by %Brix. IgG=Immunoglobulin G. ELISA=Enzyme-linked immunosorbent assay

## Discussion

An association between newborn growth rate and IgG content in serum of neonate has been shown in ruminants, such as calves and buffaloes [5,6]. Consumption of the 1^st^ day colostrum enhanced gastrointestinal development and reduced mortality in calves compared with those receiving transition milk or plain milk [8]. Therefore, early and rapid assessment of colostrum quality is essential for farm management, as this provides farmers with the necessary information to ensure the adequacy of IgG consumption by neonates. Validation of equipment is important to ensure that farmers do not eliminate good colostrum based on inaccurate results. However, choosing the cutoff threshold for standard IgG concentration may be problematic because of the methods of measurement. Our data demonstrated a strong relationship between IgG measured using ELISA and %Brix in colostrum in frozen samples as reported previously [4,21], %Brix readings did not differ between fresh or frozen samples.

Refractometers have been used for measuring the specific gravity (colostrometer), TS (%Brix), and refractive index (nD) and have been introduced to estimate IgG content in the colostrum of many species [4,14-18]. The colostrometer was invented for on-farm use and, in bovine colostrum, accurately measures IgG concentration, with results better than those using %Brix [18]. However, colostrometer is temperature sensitive [22]. Results from %Brix are not affected by multiple freeze-thaw cyclesof the sample, as previously demonstrated by a study in Jersey dairy cattle [23]. Moreover, a Brix refractometer is easy to handle, produces results quickly, and is not expensive, and the measurement of colostrum IgG is specific and reliable [21]. Only a minority of optical refractometers are calibrated to measure beyond an upper limit of 32%. Nevertheless, since%Brix reading above 32% would be regarded as high IgG content, and typical cutoff values are below this level, the tool can still be used to verify IgG levels in colostrum.

Mean IgG values in the present study were 10.6±0.6 mg/mL (range, 6.6-16.2 mg/mL), similar to those in a previous study [20]. The mean values fell within the range obtained for colostrum collected from immediately after delivery until 24 h in Murciano-Granadina goats (28.2-5.0 mg/mL) [1] but were lower than the range in Majorera goats (33.0-20.1 mg/mL) [2]. Differences in reported levels of IgG are strongly influenced by the time of collection after parturition since IgG levels have been found to decrease dramatically within an hour of parturition [1]. Therefore, when measuring by Brix refractometer and using a cutoff point, measurements must be carried out within appropriate time limits after delivery.

In the present study, the mean±SD and range of %Brix values in colostrum were, respectively, slightly higher and narrower (25.0±3.9% vs. 21.6±5.3%; 16-32% vs. 8.8-39.8%) than those in a previous study in goats [16]. We found a positive correlation between IgG measured using ELISA and %Brix (0.593), which have been previously reported using Brix or refractometer for the measurement of IgG in colostrum in many other species, including sows [13], ruminants [4,14-16,21], and in serum of horses [17]. In cattle, correlation coefficients between %Brix and colostrum IgG concentrations measured using RID have been reported as 0.64 [18], 0.79 [23], 0.71 [21], and 0.83 (the last using ELISA) [16]. In goats, the correlation coefficient between %Brix and colostrum IgG concentration measured using ELISA was 0.83 [16]. However, one study showed %Brix correlated with IgG when measured using RID but not ELISA (r=0.02) [4].

The area under the operating curve in this study was 0.987 at the cutoff point of <6.9 mg/mL IgG as determined through ELISA and %Brix. In cows, the threshold of IgG for poor-quality colostrum as determined through both RID and ELISA was given as 50 mg/mL corresponding to cutoff points for %Brix at 23% [18], 22% [21], 21% [14], 19.3% [16], and 18% [23]. Suggestions for cutoff values were based on ranges that yielded high sensitivity and specificity. However, the method of measurement affected the IgG levels. The average levels of IgG in colostrum collected a few days postpartum were 63.4 and 20.7 g/L as measured using RID and ELISA, respectively [4]. Thus, the cutoff value for IgG concentration in colostrum of goat using commercial ELISA was suggested to be 20 mg/mL [16,19]. Unfortunately, the ELISA method did not correlate well with %Brix when compared with the RID method [4]. The inconsistency in levels of IgG using ELISA suggests that the cutoff value for %Brix should be verified for each farm at which IgG measurement is performed. There is also the need to limit the amount of colostrum discarded as “low quality” to no more than 10% of the total dam population.

In our study, the %Brix cutoff threshold value is 18.5. At this point, the specificity was 100% with high accuracy (95.2%), but low sensitivity (50%). The low sensitivity was due to the limited sample size. High values for NPV and PPV suggested that good-quality colostrum would not be discarded. A cutoff value of <21.5 provided a sensitivity of 100% and a specificity of 94.7%. Although the accuracy is similar to that of a cutoff at <18.5, a lower PPV of 66.7% suggests that some good colostrum may be categorized as poor and be discarded. Thus, the appropriate cutoff point would be 18.5% at this farm. A previous study suggested that the optimal cutoff point for %Brix in goats was 20.7 equating to 53.5% sensitivity and 100% specificity [16]. Different %Brix cutoff values between 18.5% and 21.5% may be applied according to management practices, colostrum yield, or health status of the goats at each farm.

The present study showed a relationship between %Brix and fat content. It has been demonstrated previously that the fat influenced Brix value positively [24] and may affect the IgG estimation.

## Limitations of the study

A relatively small sample size was used in this study to calculate the cutoff value for %Brix, and this may have affected specificity and sensitivity. Moreover, other factors such as breed, the season of kidding, litter size, and parity of dam that affect the IgG levels [20] were not considered. Note that our cutoff values applied only to %Brix measurement of colostrum performed within 3 h of kid delivery.

## Conclusion

A Brix refractometer is an acceptable tool for evaluating poor-quality colostrum. A %Brix value of <18.5 in colostrum collected within 3 h of parturition was used as the cutoff value for poor-quality colostrum for goats on the farm used in this study.

## Authors’ Contributions

CB, ST, and KK: Contributed to the conception and design of the study, performed the statistical analysis, and wrote the manuscript. CB and SS: Contributed to data collection and laboratory analysis. MN and KK: Revised the manuscript. All authors read and approved the final manuscript.
